# Low Frequency, High Complexity: Assessing Skill Decay in Transesophageal Echocardiography Post-Simulation Training

**DOI:** 10.5811/westjem.35857

**Published:** 2025-06-25

**Authors:** Enyo Ablordeppey, Emily Terian, Collyn T. Murray, Laura Wallace, Wendy Huang, Erica Blustein, Alexander Croft, Ernesto Romo, Mansi Agarwal, Daniel Theodoro

**Affiliations:** * Washington University School of Medicine, Department of Anesthesiology, St. Louis, Missouri; † Washington University School of Medicine, Department of Emergency Medicine, St. Louis, Missouri; ‡ Washington University School of Medicine, St. Louis, Missouri. University of Chicago, Department of Emergency Medicine, Chicago, Illinois; § University of North Carolina Chapel Hill, Department of Emergency Medicine, Chapel Hill, North Carolina; || Washington University School of Medicine, Department of Biostatistics, St. Louis, Missouri

## Abstract

**Introduction:**

Resuscitative transesophageal echo (rTEE) is a promising adjunct to cardiac arrest resuscitation. However, it is a high-acuity diagnostic tool that is rarely used in this setting and its safety establishment is limited because of low occurrence. High-acuity, low occurrence skills such as rTEE during cardiac arrest inevitably decay. In this study we examined the content and percentage of rTEE skill decay following simulation-based education (SBE).

**Methods:**

Resuscitative TEE-naïve emergency physicians (EP) were trained using a combination of clinical exposure, web-based didactics, and monthly hands-on sessions with a high-fidelity rTEE simulator for four months. The COVID-19 pandemic created a natural wash-out phase where EPs did not perform any actual or SBE for six months after initial training. Unadvertised assessment of rTEE skill occurred at month 6 after rTEE training to test skill decay and at month 7 to determine the effect of spaced repetition. One year later, the EPs completed a questionnaire assessing rTEE comfort. Statistical measures were used to measure skill decay.

**Results:**

Seven EPs were individually evaluated in four domains: name recall; probe manipulation (rotation); probe manipulation (omniplane); and image acquisition adequacy. At the end of training, all participants reached a full proficiency score of 32. At month 6, the mean score was 19 of 32 (SD ±7), reflecting a 41% decay (95% confidence interval (CI) −54%, −27%; *P* < .001) for eight standard rTEE views. Following spaced repetition at month 7, the median score improved to 26 (IQR 23–30), representing a 19% decay (95% CI −35%, −4%; *P* < .02). For the three guideline-recommended views, the overall decay percentage was 26% (95% CI −36%, −16%; *P* < .001), although image acquisition skills did not show significant decay. Spaced repetition resulted in a 23% increase in mean scores (95% CI 9–37%), and the average time to obtain all eight rTEE views decreased from 7.3 minutes at month 6 to 5.7 minutes at month 7.

**Conclusion:**

After focused, proficiency-based SBE in rTEE, hands-on image acquisition skills showed the least decay compared to name recall and probe manipulation. Spaced repetition mitigated decay over one month, although not back to baseline.

## INTRODUCTION

Resuscitative transesophageal echocardiography (rTEE) is being explored as a potential tool to enhance cardiac arrest management in the emergency department (ED) by providing real-time cardiac imaging.^1^–^4^ Although preliminary evidence suggests rTEE may improve clinical decision-making, minimize interruptions to chest compressions, and offers better diagnostic images, its benefits in cardiac arrest remain preliminary and inconclusive.^5^–^7^ As rTEE skills are not typically taught in traditional emergency medicine curricula, structured training is necessary, especially post-residency, to reduce skill decay and optimize clinical use.^8^–^10^,^11^ Our department intended to incorporate TEE into cardiac arrest management, and as part of this initiative, we began training to assess the logistics of implementation, including content, time requirements, and the need for refresher training.

Skill decay, a well-documented but often overlooked issue, refers to the reduction in performance over time and is influenced by factors such as skill complexity and frequency of use.^12^,^13^ Decay is used to describe the reduction in performance from baseline across time points, with percentages indicating the relative decline in assessment scores. Many factors contribute to the rate of skill decay, including the complexity of the skill, frequency of skill repetition in the workplace, and mastery level of skill performance.^14^–^16^ While simulation-based education (SBE) improves rTEE skills in novices, less is known about long-term retention and the ideal timing for retraining.^17^–^22^ Therefore, our primary objective was to assess the decay of rTEE image acquisition skills over time and evaluate the impact of repeated SBE on skill retention.

## METHODS

### Study Design and Setting

This was a single-group, pre/post-test natural experiment study design made possible by the COVID-19 pandemic. The study took place at a quaternary-care academic center with over 300 annual cardiac arrests and 70 emergency physician (EP) faculty. Prior to the study, rTEE was not performed by EPs in our institution and endorsement to incorporate rTEE into cardiac arrest was newly disseminated from the American College of Emergency Physicians (ACEP).^5^,^23^ Eligible subjects received no prior targeted rTEE educational exposure, indicated interest in rTEE during cardiac arrest, and agreed to monthly educational exposures for a period of four months. Subjects with prior transthoracic echocardiography experience in the ED (standard at our institution) were recruited.

#### Baseline

At the outset of this study, volunteering participants had limited exposure, skills, or knowledge regarding rTEE at baseline. Due to lack of familiarity with any rTEE principles, we performed no assessment of skills as it was presumed to be negligible to meet eligibility criteria.

**Population Health Research Capsule**

What do we already know about this issue?

*Resuscitative transesophageal echo (rTEE) is a promising but underused tool in cardiac arrest resuscitation, with limited research on skill decay and safety*.

What was the research question?

*How does simulation-based education affect rTEE skill decay in emergency physicians?*

What was the major finding of the study?

*At month 6, we found a 41% decay in rTEE skills (P < .001); spaced repetition reduced decay to 19% (P < 0.02)*.

How does this improve population health?

*Improving rTEE skill retention in emergency physicians could enhance cardiac arrest outcomes through better diagnostic capabilities*.

#### Education Program

This pilot rTEE education curriculum was developed at our institution by incorporating combined elements of educational theory and exemplars of training from other specialties, such as anesthesiology and surgery.^24^–^26^ The multimodal training program was organized into clinical introduction, synchronous didactics, and simulator training.

#### Clinical Introduction and Asynchronous Didactics

Clinical rTEE introduction in the intensive care unit or operating room involved observation of clinical rTEEs with the opportunity to manipulate the probe under the supervision of clinical experts. Asynchronous didactic content was based on the Toronto General Hospital Department of Anesthesia Perioperative Interactive Education library available on its website (pie.med.utoronto.ca/rTEE/index.htm) and HeartWorks pathology modules (Inventive Medical Ltd, London, UK; now owned by MedaPhor Group).^27^

#### Monthly rTEE Simulator Criterion Based Proficiency Training

**Figure 1:**
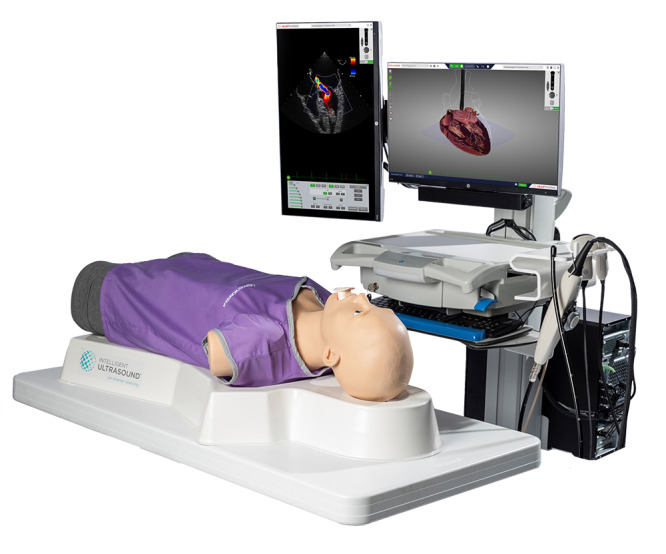
HeartWorks rTEE high fidelity simulator. *rTEE*, resuscitative transesophageal echo.

The HeartWorks rTEE was used in this study for spaced repetition (Figure 1, Image from https://www.intelligentultrasound.com/heartworks/**)**. Spaced repetition is defined as separation of training into several discrete sessions over a prolonged period with measurable intervals between training sessions. The training instructor emphasized probe manipulation and repeated acquisition of the required images performed in the same order every time. The rTEE instructor is board certified in critical care echocardiography and passed the National Board of Echocardiography Examination for Special Competence in Adult Echocardiography examination.

Eight rTEE views were selected for training based on high-yield anticipatory imaging during cardiac arrest and testing including three critical views recommended by ACEP to be used in the ED during cardiac arrest.^10^ The eight views, which represent basic images for rTEE basic certification, are as follows: 1) mid-esophageal four-chamber (ME-4C); 2) mid-esophageal aortic valve short axis (ME AV SAX); 3) mid-esophageal right ventricular inflow-outflow; 4) mid-esophageal bi-caval views; 5) mid-esophageal two-chamber (ME 2C); 6) mid-esophageal long axis view (ME LAX); 7) transgastric left ventricle short axis (TG SAX),;and 8) upper esophageal aortic long- and short-axis views. The ACEP guideline-recommended views were defined as the ME-4C, the ME LAX, and the TG SAX.

#### Skill Assessment

Manual skills and image knowledge were assessed monthly on the simulator via direct observation by an rTEE expert until criterion-based proficiency was achieved. Proficiency was defined as the errorless acquisition of eight adequate rTEE views on cue, including correct identification of anatomical targets, transducer omniplane setting, and probe rotation. The same rTEE instructor assessed proficiency each month. Training continued for four months, even after proficiency was met. By the end, all seven participants demonstrated the ability to acquire eight adequate views on demand, correctly naming each view, omniplane, and probe rotation.

We developed a checklist-based assessment tool to assess rTEE skill. The rTEE assessment score (see table, Supplemental Digital Content 1, which shows rTEE assessment variables) consisted of eight views or structures. There were four domains and the maximum score achievable was 32 points. Participants were awarded 1 point per domain (view named, probe adjustment described [rotation and omniplane], and simulated image obtained without instruction) and 0 points if elements were not obtained, respectively. No accessory recall tools, assistance, or feedback were provided during the assessment. The time to completion of the entire examination was recorded by the same training instructor supervising the study.

#### Intervention

The COVID-19 pandemic created a six-month wash-out phase to study decay where EPs were unable to use rTEE on actual patients immediately after reaching criterion-based proficiency on a rTEE simulator. No further didactics, workshops, or SBE related to rTEE occurred during the six-month wash-out period. Participants were evaluated immediately after the wash-out period (month 6 after training) and one month later (month 7) to assess the impact of spaced repetition by SBE. Following the guidance from the COVID-19 pandemic that research studies should limit face-to-face interactions during the crisis, the rTEE assessment was conducted by the same rTEE expert who provided the didactic training using the HeartWorks simulator.

### Primary Outcome

Unadvertised scoring of rTEE skill occurred after the six-month natural interruption caused by the COVID-19 pandemic. For the purposes of the study, we defined decay as the difference between full proficiency (a score of 32 points on our assessment tool) and the six-month assessment score. At month 7, scoring was repeated to determine a spaced repetition effect. Participants were unaware in advance of the assessments and were blinded to the results of the other participants. Performance results from the assessments were not reviewed with participants.

### Secondary Outcomes

#### Guideline Based Windows

We calculated the decay scores focused on three windows recommended by the ACEP published guidelines in cardiac arrest since this was the primary focus of rTEE in the ED.^10^ We also timed each participant using the simulator’s built-in timer.

#### Survey Development & Dissemination

We developed a 16-item questionnaire using a Likert scale to assess participants’ comfort with naming, describing, or performing rTEE views after SBE. One-year post-training, EPs were invited to complete the survey online, using REDCap electronic data capture tools hosted at Washington University School of Medicine. The survey, with no compensation offered, was pilot tested by two EPs (CM, DT) for relevance, clarity, and time to complete. The final survey is available in Supplemental Digital Content 2. Responses were linked to recall assessments, focusing on comfort with the eight rTEE views, SBE’s impact on comfort, and its translation to independent performance and teaching.

#### Statistical Analysis

To assess skill decay, we compared rTEE naming, probe manipulation, and simulator view assessment scores at full proficiency, month 6, and month 7. Scores at month 6 were compared to baseline proficiency, and scores at month 7 were compared to both baseline and month 6. We analyzed continuous variables using the Wilcoxon signed-rank test, with results presented as mean scores and standard deviations. Categorical variables are presented as percentages. A repeated measures analysis with fixed effects was used to evaluate differences in image acquisition success between assessments at month 6 and month 7. We compared time to complete the simulation between month 6 and month 7 using appropriate statistical methods. All tests were two-sided at a 5% significance level, conducted using SAS 9.4 (SAS Institute Inc., Cary, NC).

Survey data were exported from RedCap and analyzed with descriptive statistics. For ordinal data, we calculated median and interquartile range (IQR), and for continuous data, means with 95% confidence intervals (CI) were used. The Kendall tau correlation assessed the relationship between performance on the image acquisition test and participants’ comfort levels

The study was approved by the institutional review board. The material support was provided by departmental funding and consisted of educational website didactics and a rTEE Simulation Center. Manuscript preparation was conducted following the Consensus-Based Checklist for Reporting of Survey Studies (Table, Supplemental Digital Content 3, which shows the CROSS checklist),^28^ and STROBE^29^ guidelines (Table, Supplemental Digital Content 4, which shows the guide).

#### Sample Size

We hypothesized a 30% decline in each participant’s rTEE score using our assessment tool (four assessed domains: name recall, probe omniplane, probe rotation, and image acquisition adequacy, with a maximum possible score of 32 points) at month 6. Our rationale was that a 30% decay would reflect a relevant decline in performance requiring intervention. We conducted a Wilcoxon signed-rank test for matched pairs with 90% power and estimated that seven EPs would be needed to detect a significant effect, assuming one participant might decline re-testing.^15^,^30^,^31^

## RESULTS

A total of seven EMPs participated in the study, achieving full proficiency in February 2020, after four months of training. The participants included four females and three males, holding academic faculty appointments as clinical instructors (n=2) and assistant professors (n=5). All were proficient in transthoracic echocardiography (TTE) but novice in transesophageal echocardiography. Five participants had completed an emergency ultrasound fellowship.

### Decay Scores and Effect of Spaced Repetition

Table 1 shows the decay scores for all eight rTEE views at months 6 and 7, as well as the effect of spaced repetition. At month 0 (end of training), full proficiency is achieved, and performance score is 32/32. At month 6, there was a 41% decay in performance compared to baseline proficiency, and a 19% decay at month 7. For the three ACEP guideline-recommended rTEE views, a 26% decay in performance was observed that reduced to 8% at month 7. Spaced repetition helped reduce decay in both eight- and three-view assessments, and although statistically significant, scores did not return to baseline proficiency. In the three views, decay was most evident in naming images and recalling probe adjustments, while no decay occurred in image acquisition.

### Achievement by Domain

**Figure 2:**
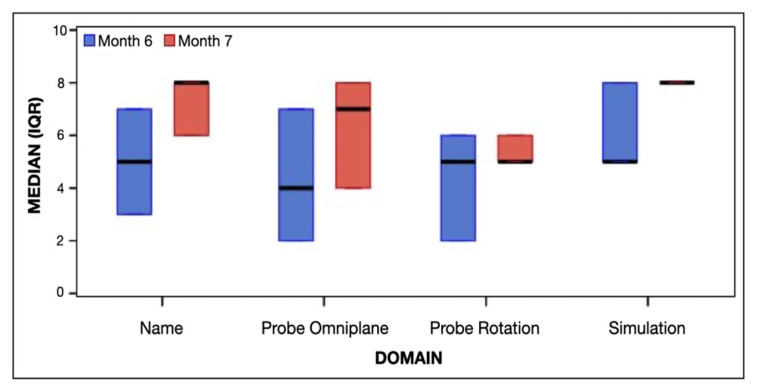
Median and interquartile ranges of achievement by domain and test month. The highest achievable score of each attempt was 32, with 8 possible points allotted to each of the four domains.

Figure 2 presents the median and IQR of scores by domain and test month. At month 6, the total median score was 20 (IQR 12–25), and at month 7, the total median score increased to 25 (IQR 23–30), although this change was not statistically significant (*P* = 0.61). While median scores improved across domains, no significant changes were observed.

### Comparison of First Attempts at Month 6 and Month 7

Table 2 compares first attempts at month 6 and month 7. The largest improvement was in name recall for the AV SAX view, with a mean difference of 0.71 (95% CI 0.26–1.17), *P* = .008. This indicates a 71% increase in correct identification at month 7. Simulation performance for the AV SAX view also improved, showing a 71% increase in mean scores (95% CI 0.26, 1.17, *P* = .008). This was the only view with statistically significant improvement. Improvements were observed for name recall and probe adjustment in other views, but these differences were not statistically significant.

### Time Decay

**Figure 3:**
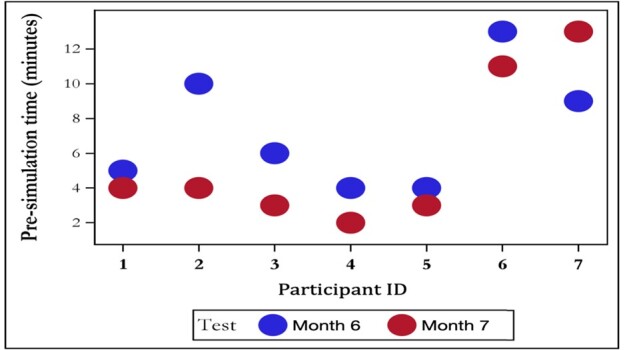
Eight view image acquisition time (minutes) during months 6 and 7 during simulation.

Figure 3 illustrates the time decay results. At month 6, the mean time for the first attempt to obtain all eight views was 7.3 minutes, decreasing to 4.6 minutes on the second attempt. At month 7, the first attempt time decreased to 5.7 minutes, and the second attempt time decreased to 4.0 minutes. However, the time decay between the first attempts at months 6 and 7 was not statistically significant (*P* = 0.12).

### Survey Results

The survey had a 100% response rate, with detailed results in the Appendix. Most participants felt simulation training effectively prepared them for rTEE during cardiac arrest, particularly in their comfort level with the three ACEP guideline views and confidence in performing rTEE. Participants were more comfortable with the simulator than human subjects and more confident with the three ACEP views compared to all eight views. Confidence in teaching others was lower. Strong correlations were found between comfort with view naming, probe manipulation, and simulation performance, particularly for omniplane position (0.73, *P* = .06 at month 6; .91, *P* = .004 at month 7).

## DISCUSSION

While several studies have assessed rTEE skill acquisition among EPs, few have examined skill decay over extended periods without retraining or clinical use.^30^,^32^–^34^,^35^ Our data highlights three key findings relevant to curriculum development for learners. First, similar to previous studies our data shows that rTEE proficiency declines by over 30% after six months without retraining (spaced repetition) or clinical exposure.^36^,^37^ This supports the notion that skill decay can occur relatively quickly without reinforcement. However, due to the lack of clinical application in our study, it is unclear whether this decay would occur in real-world settings. While prior studies suggest a wide range of skill retention patterns, our findings point to the potential need for periodic retraining in skills like rTEE procedures, where clinical opportunities may be limited.^35^,^38^,^39^,^40^ Further research is needed to determine an optimal time frame for rTEE proficiency maintenance, especially in high-acuity, low-occurrence procedures like rTEE.

Second, despite overall performance decay, participants retained the ability to perform a focused set of three key rTEE views, as recommended by guidelines. This phenomenon may be explained by participant’s motivation to focus on guideline recommendations or because overtraining (ie, training to greater standards than the three guideline recommended views) on these views helped minimize decay. While the impact of overtraining is debated, it may help preserve procedural skills like image acquisition more than cognitive tasks.^13^,^41^,^42^ These findings can inform simulation-based training curriculums, although further research is needed to understand the effects of overtraining and its long-term benefits.^13^

Third, one year after simulation training, participants showed greater confidence in obtaining three focused rTEE views (ME 4Ch, ME LAX, TG SAX) compared to all eight views. Consistently higher scores for the three views in name recall, probe adjustment, and simulation suggest that participants were more comfortable with these key views. This may be due to their similarity to familiar TTE views, which could have contributed to improved performance and confidence in acquiring and interpreting these specific views during cardiac arrest.

These results inform rTEE curriculum development, highlighting the value of simulation for skill acquisition.^9^,^29^,^37^,^38^ While one hour of simulation has shown effectiveness in previous studies, our findings suggest that extended simulator time and overtraining may be key for achieving proficiency.^18^,^27^ Our data also suggests that complex physical tasks like rTEE experience more decay in cognitive skills (eg, name recall) than in procedural skills (eg, image acquisition).^12^ Similar to other studies, we found that spaced repetition plays a role in diminishing skill decay, but further work is necessary to define optimal repetition intervals.^13^,^41^ Our pilot curriculum indicates that a didactics-simulation model, with spaced repetition, can effectively counter skill decay.

## LIMITATIONS

This study’s small sample size and single-institution design limit generalizability to other rTEE training programs. Using the same educator for training, proficiency assessments, and post-assessments may have introduced bias, particularly regarding skill decay. While participants were rTEE-naïve, their prior ultrasound experience may have led to higher skill retention, limiting generalizability to a broader EP population. The sample was also homogeneous in ultrasound knowledge; so future studies with more varied trainee populations are needed to validate these results. Our study focused on image acquisition of “normal” cardiac anatomy, and while pathology was covered in didactics, we did not assess image interpretation in clinical scenarios. The potential for training to transfer to real-world pathology identification remains unclear as clinical exposure was not evaluated. Finally, the simulator may be easier than clinical practice due to the absence of complications like probe insertion or chest compression motion. Thus, the simulator should be viewed as a complementary tool rather than a replacement for traditional, hands-on training.

## CONCLUSION

Proficiency-based overtraining on a resuscitative transesophageal echocardiography simulator demonstrated significant decay in rTEE skills after six months of non-use, although the ability to acquire focused rTEE windows persisted. Monthly spaced repetition statistically improved skill levels but did not fully reverse decay. Further studies are needed to optimize rTEE curricula, refine training intervals, and develop strategies to minimize skill decay, especially in the context of cardiac arrest management.

## Supplementary Information









## Figures and Tables

**Table 1:** Decay scores after a six-month washout and the effect of an interspaced learning intervention.

Time Interval Between Full Proficiency (score of 32) and Re-assessment	Mean Score (±SD)	Mean Score Decay^1^ (%) (95% CI)	*P*-value
**8 Views** 2			
6-month score	19 (7)	−41 (−54, −27)	<.001
7-month score (following spaced repetition)	26 (4)	−19 (−35,−4)	.02
Score following spaced repetition (7 month vs 6 month)	n/a	23 (9, 37)	.003
**3 Views**^3^ **(Guideline Recommended)**			
6-month score	9 (2)	−26 (−36,−16)	<.001
7-month score	11 (1)	−8 (−19, 2)	.10
Score following spaced repetition (7 month vs 6 month)	n/a	18 (8, 28)	.002
6-month image acquisition score^4^	3 (0)	0 (−8, 8)	1.00
6-month naming score^4^	2 (1)	−29 (−51, −6)	.02
6-month probe adjustment score^5^	4 (1)	−38 (−52, −23)	<.001

1 Negative score indicates decay,

2 Maximum score 32 (includes naming, omniplane manipulation, probe rotation, and image acquisition).

3 Maximum score 12 (includes naming, omniplane manipulation, probe rotation, and image acquisition)’

4 Maximum score 3.

5 Maximum Score 6.

*IQR*, interquartile range.

**Table 2:** Repeated measures analysis between month 6 and 7 in four domains.

	Naming		Probe Omniplane		Simulation	
		
	Mean Difference (95% CI)	*P*-value	Mean Difference (95% CI)	*P*-value	Mean Difference (95% CI)	*P*-value
1. ME 4/5 Chamber	0.29 (−0.17, 0.74)	.17	0.14 (−0.21, 0.49)	.36	100% Achievement	
2. ME AV SAX	0.71 (0.26, 1.17)	.008	0.29 (−0.17, 0.74)	.17	0.71 (0.26, 1.17)	.008
3. ME RV In-Out	0.43 (−0.07, 0.92)	.08	0.29 (−0.17, 0.74)	.17	0.14 (−0.21, 0.49)	.36
4. ME 2 Chamber/LAA	0.14 (−0.5, 0.78)	.60	0.14 (−0.5, 0.78)	.60	0.43 (−0.07, 0.92)	.08
5. ME Bicaval	0.29 (−0.17, 0.74)	.17	0.29 (−0.17, 0.74)	.17	0.14 (−0.21, 0.49)	.36
6. ME LAX	0.14 (−0.21, 0.49)	.36	0.43 (−0.07, 0.92)	.08	−0.14 (−0.49, 0.21)	.36
7. TG Mid SAX	0.14 (−0.21, 0.49)	.36	0.43 (−0.07, 0.92)	.08	100% Achievement	
8. ME Dec/Asc Aorta LAX/SAX	0.43 (−0.07, 0.92)	.08	100% Achievement		0.43 (−0.07, 0.92)	.08

Results are reported as proportions which are expressed as means (SE).

*ME*, midesophageal; *AV*, aortic valve; *SAX*, short axis; *RV*, right ventricle; *LAA*, left atrial appendage; *LAX*, long axis; *TG*, Transgastric; *Dec*, descending; *Asc*, ascending; *CI*, confidence interval.
