# Supplementary material for: Low Frequency, High Complexity: Assessing Skill Decay in Transesophageal Echocardiography Post-Simulation Training
Source: West J Emerg Med. 2025 Jun 25;26(4):1070–7. doi: 10.5811/westjem.35857 (PMC12342464; doi:10.5811/westjem.35857)
Supplement: Supplementary file 1 [file wjem-26-1070-s001.pdf]

Transesophageal Cardiac Ultrasound Simulation Recall Assessment

Name: **Select Date:** ☐ **Month 10** ☐ **Month 11**

Without prompting or feedback, the instructor asks participant to provide information listed below about TEE image names, description of how to obtain image, and actual image acquisition on simulation.

**1st attempt**		**Name the 8 Procedure Imaging Planes**	**2nd attempt**	
Achieved	Not Achieved		Achieved	Not Achieved
		1. ME 4/5 Chamber		
		2. ME AV SAX		
		3. ME RV In-Out		
		4. ME 2 Chamber (90 degrees)		
		5. ME Bicaval		
		6. ME LAX		
		7. TG Mid SAX		
		8. ME Dec/Asc Aorta LAX /SAX		

Notes:

**1st attempt**				**Describe how to obtain the 8 Procedure Imaging Planes**	**2nd attempt**			
Achieved		Not Achieved			Achieved		Not Achieved	
Omniplane	Rotation	Omniplane	Rotation		Omniplane	Rotation	Omniplane	Rotation
				1. ME 4/5 Chamber (0)-N				
				2. ME AV SAX (30)-R				
				3. ME RV In-Out (45-60)-R				
				4. ME 2 Chamber (90 degrees)-N				
				5. ME Bicaval (90)-R				
				6. ME LAX (120)-N				
				7. TG Mid SAX (0)-N				
				8. ME Dec/Asc Aorta LAX /SAX (0/90)-L				

Notes:

**1st attempt**			**Perform the 8 Procedure Imaging Planes**	**2nd attempt** *only necessary if unable to complete 1st attempt successfully		
Time	Achieved	Not Achieved		Achieved	Not Achieved	Time
			1. ME 4/5 Chamber (0)			
			2. ME AV SAX (30)			
			3. ME RV In-Out (45-60)			
			4. ME 2 Chamber (90 degrees)			
			5. ME Bicaval (90)			
			6. ME LAX (120)			
			7. TG Mid SAX (0)			
			8. ME Dec/Asc Aorta LAX /SAX (0/90)			
Total time				Total time		

Notes:
