# Supplementary material for: Low Frequency, High Complexity: Assessing Skill Decay in Transesophageal Echocardiography Post-Simulation Training
Source: West J Emerg Med. 2025 Jun 25;26(4):1070–7. doi: 10.5811/westjem.35857 (PMC12342464; doi:10.5811/westjem.35857)
Supplement: Supplementary file 2 [file wjem-26-1070-s002.pdf]

**Transesophageal Cardiac Ultrasound Simulation POST training Questionnaire**

**Name:** **Date:**

**Select responses regarding your TEE simulation training that occurred in March 2020 and the recall evaluation that occurred in October and November 2020.**

## Select your answer to the following questions

Questions 1-2 pertain to TEE Simulation Training in March 2020

1. I was comfortable obtaining TEE views on the simulator by the end of the initial simulation training in **March 2020**
   Strongly agree Agree Neutral Disagree Strongly disagree

2. I could be comfortable obtaining TEE views on a human by the end of the initial simulation training in **March 2020**
   Strongly agree Agree Neutral Disagree Strongly disagree

Questions 3-5 pertain to TEE Simulation Testing in October/November 2020

3. I was comfortable naming the TEE views during my post simulation training evaluation in **October/November 2020**
   Strongly agree Agree Neutral Disagree Strongly disagree

4. I was comfortable describing how to obtain TEE views during my post simulation training evaluation in **October/November 2020**
   Strongly agree Agree Neutral Disagree Strongly disagree

5. I was comfortable obtaining TEE views on the simulator during my post simulation training evaluation in **October/November 2020**
   Strongly agree Agree Neutral Disagree Strongly disagree

Questions 6-15 pertain to POST TEE Simulation Testing in October/November 2020

6. How many TEEs have you performed on a human since the post simulation training evaluation in **October/November 2020**
   Zero 1-3 3-5 6-10 >10

7. Of the TEEs obtained on a human, how often do you perform all 8 focused TEE views in cardiac arrest?
   Never Not usually Sometimes Most times Always

8. Currently, I am comfortable obtaining the 3 ACEP views of a focused TEE exam on a human during cardiac arrest?
   Strongly agree Agree Neutral Disagree Strongly disagree

9. Currently, I am comfortable obtaining the 8 views of a focused TEE exam on a human during cardiac arrest?
   Strongly agree Agree Neutral Disagree Strongly disagree

10. Rank each TEE view from **1 to 5** on your current level of comfort obtaining that view on a SIMULATOR.
    **Rank 1 for least comfortable and 5 for most comfortable**

    ___Midesophageal 4 chamber view
    ___Midesophageal aortic valve view
    ___Midesophageal right ventricular inflow/outflow view
    ___Midesophageal Bicaval view
    ___Midesophageal 2 chamber view
    ___Midesophageal long axis
    ___Transgastric left ventricular short axis
    ___Midesophageal aorta (short/long axis)

11. Rank each TEE view from **1 to 5** on your current level of comfort obtaining that view on a HUMAN during cardiac arrest? **Rank 1 for least comfortable and 5 for most comfortable**

    ___Midesophageal 4 chamber view
    ___Midesophageal aortic valve view
    ___Midesophageal right ventricular inflow/outflow view
    ___Midesophageal Bicaval view
    ___Midesophageal 2 chamber view
    ___Midesophageal long axis
    ___Transgastric left ventricular short axis
    ___Midesophageal aorta (short/long axis)

12. I am more likely to perform a TEE after the simulation training.
    Strongly agree     Agree     Neutral     Disagree     Strongly disagree

13. TEE Simulation training has directly impacted my confidence in obtaining TEE views during cardiac arrest
    Strongly agree     Agree     Neutral     Disagree     Strongly disagree

14. Currently, I am comfortable interpreting anatomy during a focused TEE exam on a human during cardiac arrest?
    Strongly agree     Agree     Neutral     Disagree     Strongly disagree

15. TEE Simulation training has directly impacted my confidence in interpreting TEE during cardiac arrest
    Strongly agree     Agree     Neutral     Disagree     Strongly disagree

16. How comfortable are you TEACHING someone else to obtain each view during cardiac arrest?
    **Rank 1 for least comfortable and 5 for most likely**

___Midesophageal 4 chamber view
___Midesophageal aortic valve view
___Midesophageal right ventricular inflow/outflow view
___Midesophageal Bicaval view
___Midesophageal 2 chamber view
___Midesophageal long axis
___Transgastric left ventricular short axis
___Midesophageal aorta (short/long axis)
